# Phase II clinical trial of docetaxel and trastuzumab for HER2-positive advanced extramammary Paget’s disease (EMPD-HER2DOC)

**DOI:** 10.1093/oncolo/oyae097

**Published:** 2024-06-01

**Authors:** Ikuko Hirai, Keiji Tanese, Yoshio Nakamura, Keitaro Fukuda, Takeshi Ouchi, Tetsu Hayashida, Kaori Kameyama, Takayuki Abe, Masayuki Amagai, Takeru Funakoshi

**Affiliations:** Department of Dermatology, Keio University School of Medicine, Tokyo 160-8582Japan; Department of Dermatology, Keio University School of Medicine, Tokyo 160-8582Japan; Department of Dermatology, Keio University School of Medicine, Tokyo 160-8582Japan; Department of Dermatology, Keio University School of Medicine, Tokyo 160-8582Japan; Department of Dermatology, Keio University School of Medicine, Tokyo 160-8582Japan; Department of Surgery, Keio University School of Medicine, Tokyo 160-8582Japan; Department of Pathology, Keio University School of Medicine, Tokyo 160-8582Japan; Clinical and Translational Research Center, Keio University School of Medicine, Tokyo 160-8582Japan; Kyoto Women’s University, Faculty of Data Science, Kyoto 605-8501Japan; Department of Dermatology, Keio University School of Medicine, Tokyo 160-8582Japan; Department of Dermatology, Keio University School of Medicine, Tokyo 160-8582Japan

**Keywords:** extramammary Paget’s disease, HER2, trastuzumab, docetaxel, chemotherapy

## Abstract

**Background:**

No consensus has been reached regarding the optimal chemotherapy for metastatic extramammary Paget’s disease (EMPD), a rare cutaneous adenocarcinoma, because of the lack of solid evidence from prospective trials. However, the immunohistochemical profile of EMPD reportedly resembles that of breast cancer, particularly in terms of human epidermal growth factor receptor 2 (HER2) expression, suggesting that HER2 is a promising therapeutic target for advanced HER2-positive EMPD.

**Methods:**

In this phase II single-arm trial, 13 Japanese patients received intravenous trastuzumab (loading dose of 8 mg/kg and maintenance dose of 6 mg/kg) and docetaxel (75 mg/m^2^) every 3 weeks for up to 2 years. The docetaxel dose was reduced or discontinued according to its toxicity. The primary trial endpoints were objective response rate (ORR) after 3 cycles of treatment and safety throughout the study period.

**Results:**

All 13 patients completed 3 cycles of combination therapy. The median follow-up was 27.9 months. The ORR was 76.9% (*n* = 10/13; 90% CI, 50.5-93.4). Frequently observed adverse events were neutropenia (100%), hypoalbuminemia (84.6%), and mucocutaneous infection (84.6%), all of which were well tolerated.

**Conclusion:**

The combination of docetaxel and trastuzumab demonstrated a favorable clinical effect and acceptable tolerability, which makes it a good treatment option for HER2-positive metastatic EMPD (ClinicalTrials.gov Identifier: UMIN000021311, jRCTs031180073).

## Teaser

This first prospective study provides new evidence for the treatment of metastatic extramammary Paget’s disease, which had no consensus on the optimal chemotherapy.

**Lessons learned**

- Docetaxel-trastuzumab combination for HER2-positive extramammary Paget’s disease demonstrated an objective response rate of 76.9% and a manageable safety profile.
- This first prospective study provides new evidence for treatment of metastatic extramammary Paget’s disease which had no consensus on the optimal chemotherapy.

## Discussion

Extramammary Paget’s disease (EMPD) is an extremely rare intraepidermal adenocarcinoma that principally affects the genital and axillary regions of the elderly. The prognosis of non-metastatic EMPD is generally favorable with surgical resection; however, that of EMPD with lymph node and visceral metastasis is poor.

Benefits of cytotoxic drug-based chemotherapy regimens including single-agent docetaxel and taxane and/or platinum-based combination regimens have been reported for the treatment of metastatic EMPD. However, the benefits of these regimens have a low level of evidence because all data are based on individual case reports or the results of small retrospective analyses.

Although the pathogenesis of EMPD remains poorly understood, its immunohistochemical profile reportedly resembles that of breast cancer, particularly in terms of human epidermal growth factor receptor 2 (HER2) expression. In a previous study, 37.1% (13/35 cases) of metastatic lesions exhibited HER2 gene amplification.^1^ HER2 has also been described as a therapeutic target. In breast cancer, the combination of HER2 inhibitors and taxanes is now an established first-line treatment. These findings suggest that HER2 is a promising therapeutic target for advanced HER2-positive EMPD. In fact, several case reports have described the efficacy of trastuzumab as monotherapy or in combination with taxanes.^2-4^ However, its utility for the treatment of HER2-positive EMPD has not yet been evaluated in a clinical trial. Therefore, we conducted the present phase II trial to evaluate the efficacy and safety of trastuzumab/docetaxel combination treatment for HER2-positive metastatic EMPD.

**Figure 1.**
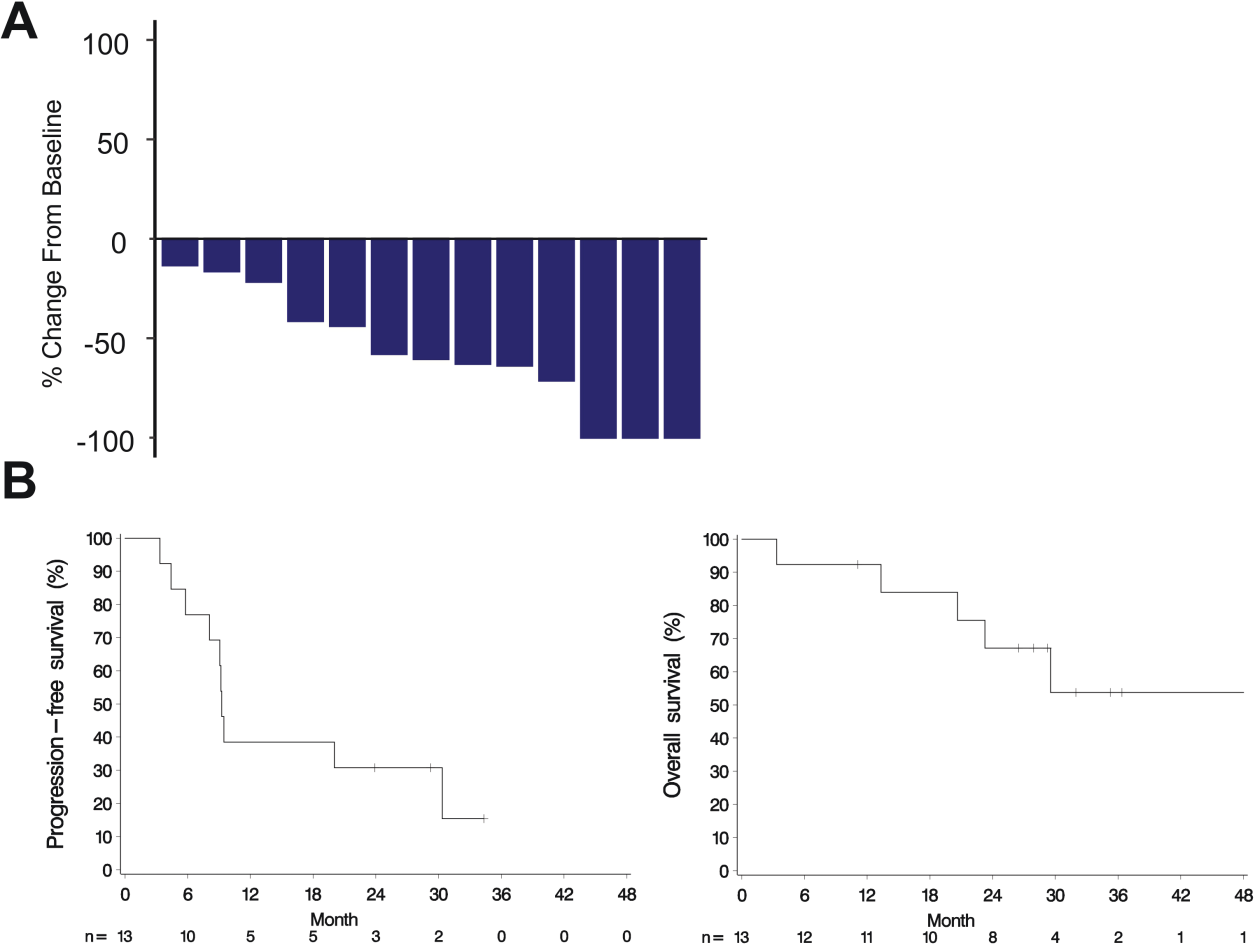
Clinical responses to trastuzumab and docetaxel in patients with advanced HER2-positive extramammary Paget’s disease. (A) Waterfall plot: relative change (%) in the sum of the diameters of target lesions at the primary endpoint after 3 cycles compared with baseline. (B) Estimated progression-free survival (left panel) and overall survival (right panel) (Kaplan-Meier plots).

In the study, 3 cycles of combination treatment resulted in an excellent response. The primary endpoint was met, with the objective response rate reaching 76.9% (90% CI, 50.5-93.4) and a disease control rate of 100% (Figure 1). Treatment efficacy was also demonstrated in patients who had failed previous chemotherapy regimens, including docetaxel monotherapy. At the data cutoff point (the median follow-up period was 27.9 months (range 3.4-49.3 months)), the mean and median PFS were 15.1 and 9.3 months, respectively and the median overall survival was not reached.

This study showed a manageable safety profile, with 2 recovered serious adverse events (AEs), one recovered AE that led to treatment discontinuation, and no treatment-related deaths. Most AEs occurred during the combination therapy, not during trastuzumab monotherapy.

The present study had several limitations. First, the number of patients was too small to assess the true safety and efficacy of docetaxel and trastuzumab combination therapy. Second, this was a single-arm study, preventing demonstration of the superiority of docetaxel and trastuzumab combination therapy over docetaxel alone. Third, this study only included Japanese patients with EMPD; ethnic variation should be considered when interpreting the results. Nevertheless, our results indicate that docetaxel and trastuzumab combination therapy can be a good treatment option for the management of HER2-positive metastatic EMPD.

## Trial Information

**Table:** 

Disease	Extramammary Paget’s disease
Stage of disease/treatment	Metastatic/advanced
Prior therapy	No designated number of regimens
Type of study	Phase II, single arm
Primary endpoint	Objective response rate after 3 cycles of treatment, safety throughout the study period
Secondary endpoints	Overall survival, progression-free survival, duration of overall response
Investigator’s analysis	Active and should be pursued further

## Additional details of endpoints or study design

### Study design

This study was a single-arm, open-label phase II clinical trial of trastuzumab and docetaxel administration for patients with HER2-positive metastatic EMPD. It was conducted as an “advanced medicine” trial authorized by the Japanese Ministry of Health, Labour, and Welfare. The study protocol was approved by the Keio Certified Review Board (CRB3180017). The cutoff point for the reported data was 28.6 months after enrollment of the last patient.

### Study population and eligibility criteria

Patients aged ≥20 years who met the following key criteria were eligible for this study: histopathologically diagnosed EMPD; HER2-positive EMPD, which was defined as HER2 immunohistochemistry (IHC) 3 + or HER2 IHC 2 + with confirmation of HER2 gene amplification by in situ hybridization, based on the HER2 testing algorithm for breast cancer of the American Society of Clinical Oncology/College of American Pathologists guideline; measurable metastatic lesions according to the Response Evaluation Criteria in Solid Tumors (RECIST) 1.1; Eastern Cooperative Oncology Group (ECOG) performance status of 0 or 1; left ventricular ejection fraction ≥ 50%; neutrophil count ≥ 1500/mm^3^; platelet count ≥ 100 000/mm^3^; hemoglobin level ≥ 10 g/dL; period > 4 weeks from the start of the previous treatment; and no evidence of active cardiac dysfunction or active brain metastases.

### Study schedule and intervention

Trastuzumab (loading dose of 8 mg/kg and maintenance dose of 6 mg/kg) and docetaxel (75 mg/m^2^) were intravenously administered every 3 weeks for one cycle. The dose of docetaxel could be reduced to 55 mg/m^2^ depending on its toxicity. If toxicity was observed even after dose reduction, the docetaxel was discontinued and protocol treatment was continued as trastuzumab monotherapy. Treatment was continued for up to 2 years, as long as the patient continued to benefit from treatment and did not meet the criteria for study discontinuation, including disease progression.

Laboratory examination and assessment of the ECOG performance status were performed at baseline, in every treatment cycle, and at the time of treatment discontinuation. The left ventricular ejection fraction was measured at baseline, every 3 cycles, and at treatment discontinuation to assess cardiac safety. All adverse events (AEs) were followed up to 4 weeks after the cessation of treatment. Patient safety was also monitored by an independent data and safety monitoring committee. The tumor response was assessed by computed tomography or magnetic resonance imaging at baseline and every 3 treatment cycles until treatment discontinuation according to RECIST 1.1. If a patient discontinued the trial treatment without disease progression, monitoring of the disease status was continued by tumor imaging until disease progression, death, or initiation of new anticancer therapy, whichever occurred first.

### Endpoints

The primary endpoints were the ORR after 3 cycles of treatment based on RECIST 1.1 and safety throughout the study period using common terminology criteria for adverse events version 4.0. The secondary endpoints were overall survival (OS), progression-free survival (PFS), and the duration of overall response (DOR). OS was defined as the time from treatment initiation to death, PFS was defined as the time that elapsed between treatment initiation and first progression or death of any cause, and DOR was defined as the length of time that a tumor remained in partial response (PR) or complete response (CR) without spread or growth.

### Evaluation of tumor biomarkers

The serum level of carcinoembryonic antigen (CEA) was analyzed using an Elecsys CEA Assay (Roche Diagnostics). The serum level of cytokeratin 19 fragment 21-1 (CYFRA 21-1) was analyzed using an ARCHITECT CYFRA 21-1 assay (Abbott Laboratories). Both levels were assessed at baseline and after 3 cycles, and the cutoff values were 5.0 and 3.5 ng/mL, respectively.

### Statistical analysis

Statistical analyses and reporting were conducted in accordance with the Consolidated Standards of Reporting Trials guidelines, with the primary analyses based on the intent-to-treat principle. All efficacy analyses were based primarily on the full analysis set, which included all patients who had received at least one dose of the study drugs. The primary endpoint for efficacy evaluation pre-specified in the protocol and in the statistical analysis plan was the ORR after 3 treatment cycles. A sample size of 13 patients was required to achieve 70% power, assuming that the expected ORR was 65% in a binomial exact test (null hypothesis: ORR ≤ 35%) with a one-sided 5% alpha level. If the lower bound of the Clopper-Pearson 90% CI for the ORR was >35%, the null hypothesis was rejected. For OS and PFS, the Kaplan-Meier method was used to estimate survival functions. All safety analyses were also based primarily on the full analysis set, which included all patients who had received at least one dose of the study drugs. All statistical analyses were performed using SAS 9.4 (SAS Institute) or SPSS Statistics 26.0 (IBM Corp.). A one-sided *P*-value < .05 was considered statistically significant in the primary endpoint analysis, and the significance level was a 2-sided *P*-value of .05 in all other analyses.

## Drug Information

**Table:** 

Generic/working name	Trastuzumab/docetaxel
Company name	Chugai Pharmaceutical Co., Ltd./ Elmed Co., Ltd. (formerly Elmed Eisai Co., Ltd.)
Drug type	Monoclonal antibody/cytotoxic agent
Drug class	An HER2-specific humanized monoclonal antibody/taxane
Dose	Trastuzumab (loading dose of 8 mg/kg and maintenance dose of 6 mg/kg)/ docetaxel (75 mg/m^2^)
Route	iv
Schedule of administration	Day1 of a 3-week cycle

## Patient Characteristics (*N* = 13 − Adenocarcinoma)

**Table:** 

Characteristic	No. of patients, *n* (%)
Median age, years (range)	68 (47-79) years
Sex	
Male	7 (53.8)
Female	6 (46.2)
PS	
0	12 (92.3)
1	1 (7.7)
No. of received pretreatment chemotherapies	
None	10 (77.0)
One regimen	2 (15.4)
Two regimens	1 (7.7)
Metastatic site at the initiation of the study	
Regional LNs	4 (30.8)
Distant LNs	12 (92.3)
Lung	3 (23.1)
Bone	1 (7.7)
Liver	3 (23.1)
HER2 status	
IHC 2 + with *HER2* gene amplification	6 (46.2)
IHC 3+	7 (53.8)

## Primary Assessment Method

**Table:** 

Title	Objective response rate after 3 cycles of treatment, safety throughout the study period
Number of patients screened	32
Number of patients enrolled	13
Number of patients evaluable for toxicity	13
Number of patients evaluated for efficacy	13
Evaluation method	RECIST 1.1
Response assessment, CR	5 (38.5%)
Response assessment, PR	5 (38.5%)
Response assessment, SD	3 (23.1%)
Response assessment, PD	0 (0%)
Median duration assessment, PFS	9.3 months (95% CI: 5.8-30.4 months)
Median duration assessment, OS	The median OS and its 95% CI could not be estimated because the estimated OS at the last time point of 49.3 months was >50%.
Response duration	12.8 months (95% CI: 1.4 to ≥28.5 months)
Duration of treatment	The median number of treatment cycles for combination therapy was 7 (range, 3-21)

## Outcome notes

### Tolerability and AEs

**Figure 2.**
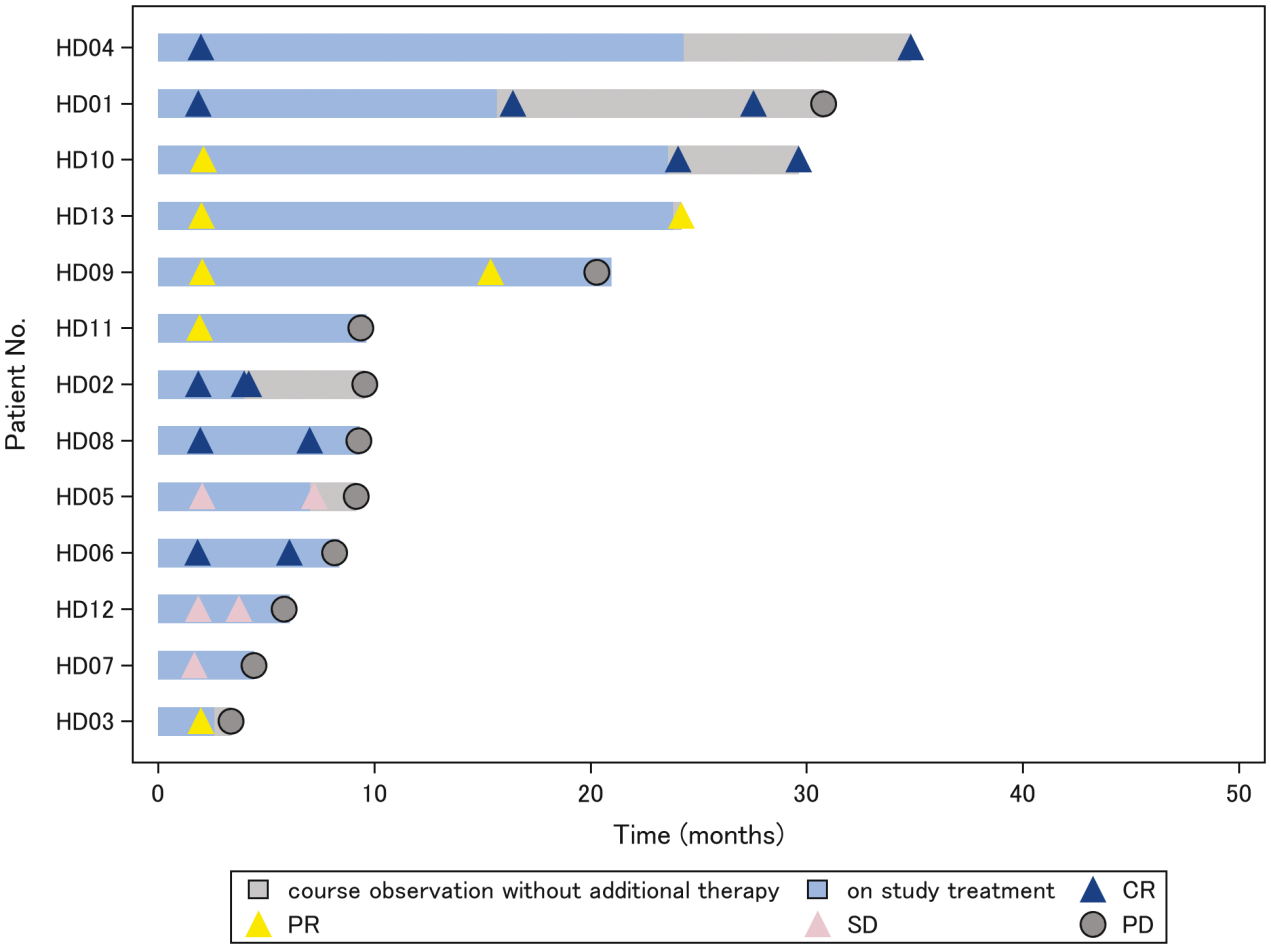
Durability of tumor regression. The study treatment exposure and its response duration are shown. Gray bars indicate treatment course observation with no additional therapy.

All 13 enrolled patients were assessed for tolerability and toxicity. All of them completed 3 cycles of protocol therapy without treatment discontinuation or treatment-related death. The protocol therapy was further continued in 12 patients, including 5 patients who received treatment over 1 year (Figure 2). The median number of treatment cycles for combination therapy was 7 (range, 3-21). The docetaxel dose was reduced in 10 patients who developed neutropenia, 7 of whom discontinued the docetaxel and continued treatment with trastuzumab monotherapy. A serious AE other than disease progression was observed in 2 patients: febrile neutropenia associated with pneumonitis in one patient and grade 3 cellulitis under neutropenia in the other patient. These patients recovered with administration of antibiotics and granulocyte colony-stimulating factor. Treatment discontinuation because of a study drug-related AE occurred in one patient who received 5 cycles of combination treatment and developed grade 1 macular edema, which resolved after treatment cessation. No treatment-related deaths occurred.

The most frequently observed AEs were hematotoxicities, including neutropenia (grade 4, *n* = 12; grade 2, *n* = 1) and anemia (grade 3, *n* = 2; grade 1 or 2, *n* = 7) (Table 1). Neutropenia was well managed by administration of granulocyte colony-stimulating factor and was present during combination treatment but not during trastuzumab monotherapy. Other commonly observed AEs were hypoalbuminemia, mucocutaneous infection under neutropenia (84.6%), and alopecia (76.9%). Most of these AEs were mild or moderate (grade 1 or 2). Two patients developed grade 3 cellulitis of the lower extremities.

**Table 1.** Incidence of adverse events in 13 patients.

Adverse events	*n* (%)		
Grade ≥ 3			
	Any grade	Grade 3	Grade 4
Hematologic toxicity			
Leukocytopenia	13 (100)	8 (61.5)	5 (38.5)
Neutropenia	13 (100)		12 (92.3)
Febrile neutropenia	1 (7.7)	1 (7.7)	
Anemia	9 (69.2)	2 (15.4)	
Non-hematologic toxicity			
Diarrhea	5 (38.5)	1 (7.7)	
Pulmonary edema	1 (7.7)	1 (7.7)	
Hypertension	2 (15.4)	2 (15.4)	
Syncope	1 (7.7)	1 (7.7)	
Urinary tract obstruction	1 (7.7)	1 (7.7)	
Mucocutaneous infection	11 (84.6)	2 (15.4)	
Arthralgia	1 (7.7)	1 (7.7)	
Grade 1/2* (*reported in ≥20% of patients)			
Alopecia	10 (76.9)		
Rash	5 (38.5)		
Nausea	6 (46.2)		
Gastralgia/gastritis	5 (38.5)		
Constipation	4 (30.8)		
Hypoalbuminemia	11 (84.6)		
Anorexia	4 (30.8)		
Pyrexia	3 (23.1)		
Peripheral edema	3 (23.1)		
Weight increased	5 (38.5)		
Aspartate aminotransferase increased	4 (30.8)		
Peripheral neuropathy	6 (46.2)		
Upper respiratory infection	7 (53.8)		
Pneumonitis	3 (23.1)		

### Efficacy

The full analysis set showed an ORR of 76.9% (*n* = 10/13; 90% CI, 50.5-93.4) after 3 cycles of combination treatment, including 5 (38.5%) patients with CR and 5 (38.5%) patients with PR (Figure 1). The lower bound of the 90% CI was greater than the prespecified threshold of the ORR (35%), and the primary study hypothesis (ORR > 35%) was demonstrated. Stable disease was seen in 3 (23.1%) patients, and the disease control rate was 100%. All 13 patients benefited from treatment without being affected by baseline characteristics such as age, number of prior systemic chemotherapy treatments, HER2 status, tumor spread, or tumor burden. In further analysis, the serum levels of CEA and CYFRA 21-1 were evaluated because their dual assessment is reportedly useful for evaluating the treatment response of metastatic EMPD.^5^ The serum level of either or both indices was elevated at baseline in all 13 patients and decreased with clinical response (Table 2).

**Table 2.** Relationship between response and transition of serum levels of carcinoembryonic antigen (CEA) and cytokine 19 fragment 21-1 (CYFRA 21-1).

Case no.	Treatment response after 3 cycles	CEA (ng/mL)		CYFRA 21-1 (ng/mL)	
		Baseline	After 3 cycles	Baseline	After 3 cycles
1	CR	178.6	21.3	3.4	2.3
2	CR	51.2	6.6	5.6	2.2
3	PR	9.4	6.4	109.0	3.7
4	CR	12.7	4.2	8.1	0.8
5	SD	8.7	6.5	8.0	2.2
6	CR	22.5	1.6	8.4	2.4
7	SD	150.9	3.0	28.8	1.3
8	CR	27.9	2.0	50.2	3.0
9	PR	5.1	1.7	3.3	0.8
10	PR	17.9	2.0	5.8	1.8
11	PR	68.2	4.3	8.2	1.1
12	SD	1.2	2.0	5.2	1.5
13	PR	226.9	7.9	10.8	2.0

The cutoff values; CEA 5.0 ng/mL, CYFRA21-1 3.5 ng/mL. All the cases with HER2 IHC 2 + in this study had *HER2* gene amplification.

Abbreviations: CR, complete response; LNs, lymph nodes; PR, partial response; RR, response rate; SD, stable disease.

After assessing the primary efficacy endpoint, all but one patient continued the study treatment. Of the 10 patients with PR or CR, 3 (30%) continued to show an initial response at the data cutoff point (Figure 2). Three patients completed the 2-year study treatment with durable responses. The median PFS was 9.3 months (95% CI, 5.8-30.4 months), and the median DOR was 12.8 months (95% CI, 1.4 to ≥28.5 months) (Figure 1). The median OS and its 95% CI could not be estimated because the estimated OS at the last time point of 49.3 months was >50% and the 3-year survival rate was 53.7% (95% CI, 20.1-78.6) (Figure 1).

## Assessment, Analysis, and Discussion

**Table:** 

Completion	Study completed
Investigator’s assessment	Active and should be pursued further

No consensus on the optimal chemotherapeutic regimen has yet been attained for the treatment of metastatic EMPD, mainly because of the rarity of the disease and the lack of well-designed clinical trials. Consequently, there is a significant unmet medical need for the treatment of metastatic EMPD. To address this issue, we designed the present study to not only evaluate the benefit of the combination of docetaxel and trastuzumab but also to establish an evidence base for the use of chemotherapy for EMPD. Although the study should have ideally been a 2-arm comparison of docetaxel monotherapy and combination therapy, it was designed as a single-arm study because metastatic EMPD itself is rare and HER2-positive cases are even rarer.

The results of the analysis of efficacy data in the present study are superior to those of 3 previous retrospective studies of docetaxel monotherapy with different dosing regimens; the response rates of 58.3%, 31.8%, and 35.7%, respectively with median PFS of 6.0-9.0 months, although it should be noted that these studies were conducted in small groups and did not examine the HER2 expression status.^6–8^ The synergistic effects of combining molecularly targeted agents with cytotoxic agents have been reported in a variety of malignancies. Trastuzumab has also shown consistent synergy with docetaxel in HER2-positive breast cancer cells across a broad range of dosing regimens.^9^ Thus, targeting HER2 in combination with docetaxel will benefit patients with HER2-positive metastatic EMPD by prolonging their prognosis.

Regarding the results of the analysis of safety data, most AEs occurred during the combination therapy, not during trastuzumab monotherapy. Although the AE profile could not be compared between combination therapy and docetaxel monotherapy, a pivotal randomized trial of HER2-positive breast cancer (M77001study) that compared combination therapy and docetaxel monotherapy showed little difference in the incidence and severity of AEs between the 2 groups.^10^ The vast majority of AEs in the present study are assumed to have been due to docetaxel. AEs with a reported association with trastuzumab, including cardiac dysfunction, were not observed in this study.^11^ Because the present study involved a limited number of patients, further accumulation of cases will be necessary to assess the toxic effects of trastuzumab in patients with metastatic EMPD.

The incidence of hematological toxicity was higher in this study than in a breast cancer trial (CLEOPATRA study), which used the same doses of docetaxel and trastuzumab as used in the present study.^12^ Although grade ≥ 3 neutropenia was observed in 45.8% of patients in the CLEOPATRA study, grade 4 neutropenia was present in 92.3% of patients in the present study. One possible reason for this discrepancy may be the difference in the patients’ ages in these 2 trials. The median age of the patients in the CLEOPATRA study was 54 years, and that of the patients in the present study was 68 years. A prospective study of triweekly docetaxel administration showed an increased risk for hematotoxicity in an elderly population because of bone marrow sensitivity.^13^ Another possible reason for the discrepancy in hematological toxicity is the racial difference between studies, which may affect the optimal dose of docetaxel. Currently, 75 mg/m^2^ is commonly administered at 3- to 4-week intervals in global clinical trials, and the rationale for dose selection in the present study was based on this evidence.^12,14–16^ However, several retrospective studies of EMPD have shown efficacy at a dose of 60 mg/m^2^.^2,3^ Thus, the optimal starting docetaxel dose of <75 mg/m^2^ should be further examined for Japanese patients with metastatic EMPD, particularly elderly patients when administered in combination with trastuzumab.

This study demonstrated a favorable clinical effect of docetaxel and trastuzumab combination therapy for the treatment of HER2-positive metastatic EMPD. Further trials that include larger populations are necessary to establish the true safety and efficacy of this treatment.

## Data Availability

The data underlying this article will be shared upon reasonable request to the corresponding author.

## Additional information

Takeru Funakoshi is the Principal investigator.
